# Heavy Metals in River Sediments: Contamination, Toxicity, and Source Identification—A Case Study from Poland

**DOI:** 10.3390/ijerph191710502

**Published:** 2022-08-23

**Authors:** Mariusz Sojka, Joanna Jaskuła

**Affiliations:** Department of Land Improvement, Environmental Development and Spatial Management, Faculty of Environmental Engineering and Mechanical Engineering, Poznań University of Life Sciences, Piątkowska St. 94, 60-649 Poznań, Poland

**Keywords:** river, sediments, heavy metals, natural processes, anthropogenic sources, PMF

## Abstract

This study investigated the spatial distribution, contamination, potential ecological risks and quantities of pollutant sources of six heavy metals (HMs) in sediments of 47 rivers. The catchments of the investigated rivers are situated in Poland, but some of them are located in Slovakia, the Czech Republic, and Germany. Cluster analysis was applied to analyze the spatial distribution of Cd, Cr, Cu, Ni, Pb, and Zn in river sediments. Moran *I* and Getis-Ord $G_{i}^{*}$ statistics were calculated to reveal the distribution pattern and hotspot values. Principal component analysis (PCA) and positive matrix factorization (PMF) were used to identify pollution sources. Furthermore, geochemical indices and sediment quality guidelines allowed us to assess sediment contamination and potential toxic effects on aquatic biota. The results showed that in 1/3rd of the rivers, the HM pattern and concentrations indicate sediment contamination. The EF, PLI, and MPI indices indicate that concentrations were at a rather low level in 2/3rd of the analyzed rivers. Only in individual rivers may the HMs have toxic effects on aquatic biota. Spatial autocorrelation analysis using the Moran *I* statistic revealed a random and dispersed pattern of HMs in river sediments. PCA analysis identified two sources of HMs’ delivery to the aquatic environment. Cr, Cu, Ni, Pb, and Zn originate from point and non-point sources, while Cd concentrations have a dominant natural origin. The PMF identified three sources of pollution. Among them, urban pollution sources are responsible for Cu delivery, agricultural pollution for Zn, and industrial pollution for Ni and Cr. Moreover, the analysis showed no relationship between catchment land-use patterns and HM content in river sediments.

## 1. Introduction

Heavy metals (HMs) in aquatic environments are present as a result of natural rock weathering processes and are also contributed from anthropogenic sources. Studies indicate that the natural biogeochemical cycle of HMs has been perturbed by anthropogenic activities [1,2,3,4,5]. Nowadays, the presence of HMs in the aquatic environment is mainly associated with anthropogenic sources [6]. In general, there is always a considerably greater pool of HMs in sediments that have a distinct anthropogenic origin [7]. Riverine sediments are highly prone to heavy metal contamination [8]. Anthropogenic HMs are generally present in higher concentrations and may have toxic effects on aquatic biota [9]. HMs can be incorporated into the food chain [10] and then potentially impact human health [11]. Major sources of pollution include agriculture, industry, domestic and miscellaneous [9,12,13]. The largest contributors to heavy metals are industrial activities [14]. In addition, the water environment is being polluted as a result of rapid urbanization [15,16,17]. Rapid urbanization has led to increased stress on rivers including impacts on water quality [18,19], eutrophication and overgrowth processes [20], floods [21], and pollution of sediments with heavy metals [22,23,24]. Additionally, agricultural intensification can be a serious problem from the perspective of aquatic pollution by HMs [25]. In general, the results of many studies show that HM concentrations can be ranked based on land use characteristics as follows: industrial region > urban region> agricultural region > natural fields [26].

HMs have low solubility in water, and are easily absorbed in sediments. Sediments are the sink for heavy metals in aquatic environments [27]. Therefore, the transport of HMs in the river system is primarily related to the sediments, especially their texture and organic matter content. HMs accumulated in river sediments as a result of fluvial processes move continuously with the river course. The accumulation and spatial distribution of HMs in river sediments are primarily controlled by physical, chemical, hydrological, and hydraulic factors [28]. Fluvial processes in the river course and floodplain alter the amount and rate of HM accumulation in sediments and the extent of downstream transport [28,29]. The HM concentration in river sediments increases from upstream to downstream [25,30]. A special role in HM transport in aquatic systems is played by lakes [31] and reservoirs [32]. The presence of lakes, reservoirs, and hydraulic structures on the river course disrupts the transport of sediments, and thus, the transport of HMs [33,34,35].

The concentration of HMs in the river sediments is related to the cumulative impact of various pollution sources and natural processes related to geological structure and climatic conditions [11,36,37], characteristics of pollution sources (point and non-point) [38], their location and land use structure [39]. In general, each catchment has a unique pattern for delivering HMs to water, driven by the type and distribution of pollution sources and emission rates [40]. Land use plays a vital role in water and sediment pollution [38,41,42]. Liu et al. [43] indicate that in addition to primary land-use types, landscape diversity and structure are important to HM supply. As a result of overlapping natural and anthropogenic impacts and fluvial processes within even the same river, HM concentrations in river sediments can vary considerably [22,44,45].

The identification of HM sources is critical in environmental studies. Sediments have become effective indicators of contamination in aquatic ecosystems [8]. HMs can preserve in sediments as a free form or variety of chemical forms in sediments, which can be related to the active substances they combine with organic matter, sulfide, carbonate, and oxy-hydroxide. Identification and quantitative and qualitative (fingerprint) description of pollution sources are necessary to determine the HMs load to waters [46]. Identification of sources of HMs in the aquatic environment is very challenging due to their overlapping impacts [47], characteristics and location. Whereas pollution point sources are relatively easy to identify and control, non-point sources are definitely a greater problem. Many studies deal with the identification of HMs sources in water and sediments [48,49,50]. Usually, scientists have large sets of measurement data, and the identification of HM sources is usually performed indirectly using various statistical methods. Among them, the most frequently applied is principal component analysis (PCA). PCA reduces the dimensionality of large datasets and improves interpretability [51]. Another statistical method used to identify sources of aquatic pollution is positive matrix factorization (PMF) [52,53,54], which is used to qualitatively analyze the impact of pollution sources [55]. The PMF method has been successfully used many times to identify sources of heavy metal contamination of rivers’, lakes’ and estuaries’ sediments [44,46,53,56,57,58,59]. Furthermore, the PMF method has also been used to identify pollution sources in aquatic environments by polycyclic aromatic hydrocarbons [60,61], synthetic organofluorine chemical compounds [62], dioxins [63,64], brominated diphenyl ethers [65] and the sources of organic carbon in sediments [66]. Additionally, PMF has been used successfully to identify sources of air [67,68,69,70,71,72] and soil pollution [73,74,75]. PMF offers advantages in comparison to other chemometric methods for identifying pollution sources [76]. PMF allows the analysis to include sampling sites with missing data or below the detection limit, which are assigned arbitrary concentrations and, consequently, high uncertainties [65]. PMF determines the connection between pollution sources and environmental quality using the principle of chemical substance balance [77]. Xia et al. [78] demonstrated the efficacy of PMF for identification of metal sources on the watershed scale. Another efficient method to identify pollution sources and quantify their impact is a combined method that links principal component analysis and multiple linear regression (PCA-MLR). For the PCA-MLR model, PCA transforms the original data into a set of linearly independent variables, and MLR is used to analyze the composition of the source [79]. PCA-MLR can solve both qualitative and quantitative pollution source identification problems [80,81]. PCA-MLR has been used for the prediction of chlorophyll-a concentration [82,83] and suspended sediment yield [84]. There are also other statistical methods used to identify sources of pollution such as conditional inference tree (CIT) [85]. Moreover, artificial neural network methods, including Kohonen’s self-organizing map, are also used to reduce and organize the structure of large datasets [6]. On the other hand, a completely different issue is the spatial presentation of environmental pollution using geostatistical methods [60]. Furthermore, geographically weighted regression is used to identify the spatially varying relationship between land-use types and total heavy metal concentrations in sediment [86]. Very often, several statistical methods are used to analyze the results. Such an approach enables one to choose the optimal statistical method from the perspective of the analyzed problem and compares the results obtained by other methods [57]. Most commonly, studies present a parallel application of basic methods such as correlation analysis in combination with PCA and PMF methods [47,87] or PCA-MLR and PMF methods [88].

The main objectives of this study were to (1) identify the statistical characteristics and compositions of heavy metals, (2) analyze sediment pollution by HMs and their potential toxic effect, (3) analyze the spatial patterns of heavy metal concentrations in river sediments and (4) conduct qualitative and quantitative analyses of the main pollution sources affecting heavy metal concentrations and spatial variability. The results of qualitative and quantitative identification of pollution sources across Poland are presented in this paper for the first time. Moreover, it is the first study to present spatial patterns of HM concentrations in river sediments on such a scale, using a GIS information system and statistical analysis tools.

## 2. Materials and Methods

### 2.1. Study Area

**Figure 1:**
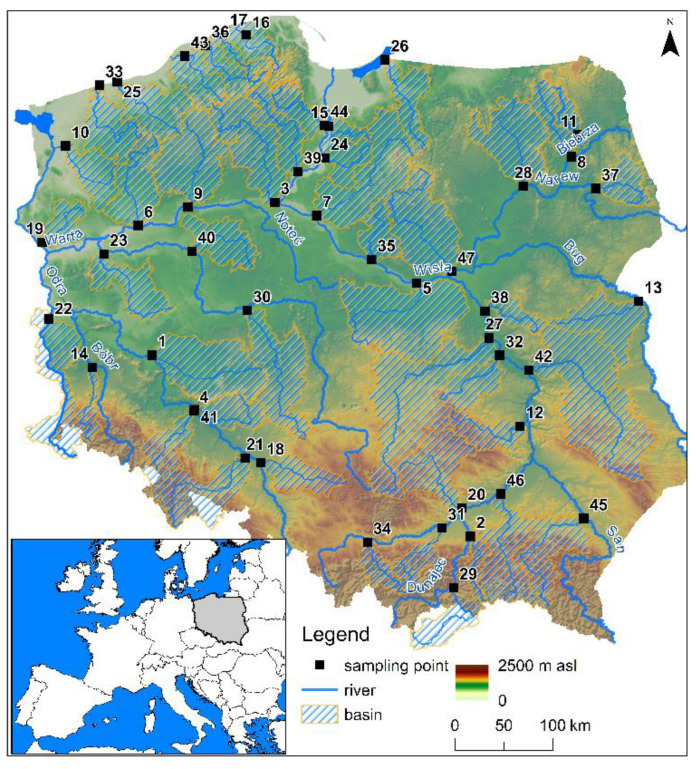
Study area location. The numbers of sampling points are listed in the Appendix A.

A total of 47 sampling points were selected for analysis of HM concentrations in river sediments. The analyzed catchments are located across the whole territory of Poland (Figure 1). Furthermore, three of them have an international character. The Poprad river basin (No. 29) is located in Poland and Slovakia, the Nysa Klodzka river basin (No. 21) is located in Poland and the Czech Republic, and the Nysa Luzycka river basin (No. 22) is located in Germany and the Czech Republic. The easternmost catchment area is the Krzna River catchment (No. 13), the westernmost is the Nysa Luzycka River catchment area (No. 22), the northernmost is the Leba River catchment area (No. 16), and the southernmost is the Poprad River catchment area (No. 29). A detailed summary of the sampling points and river names is presented in the Appendix A.

The catchments area ranges from 809.2 km^2^ (Lupawa River—No. 17) to 10,453.8 km^2^ (Wieprz River—No. 42) (Table 1). The total area of the studied catchments is 141,972.70 km^2^, which represents about 45% of the total area of Poland. The catchments represent regions with diverse climatic and hydrological conditions, topography and land cover, and human pressures associated with industrial and agricultural activities. In Poland, average annual temperatures range from 6.5 °C in the northeast to 8.5 °C in the west, with a mean value of about 7.8 °C. In the south of Poland in the mountain regions, the average annual temperatures range from 5.0 to 7.0 °C. The average annual precipitation in Poland is about 630 mm. However, the annual precipitation in the central part of Poland is about 500 mm, while in the mountain regions, southeastern Poland exceeds 1500 mm. Unit outflow in Poland is only 5.5 dm^3^·s^−1^·km^−2^ on average. The highest values of unit outflow occur in the mountainous catchments of southern Poland (50 dm^3^·s^−1^·km^−2^), while in the lowland catchments of central Poland, unit outflow reaches 2 dm^3^·s^−1^·km^−2^. Detailed catchment characteristics regarding topography, hydrographic network patterns and land cover are presented in the Appendix B.

### 2.2. Sediment Sampling and Chemical Analysis

This paper presents Cd, Cr, Cu, Ni, Pb, and Zn concentrations in the sediments of 47 rivers. The data were provided by the Chief Inspectorate of Environmental Protection in Poland. Chemical analyses of river sediments are carried out within the framework of the State Environmental Monitoring. The analysis was carried out in 2017. Chemical analysis was performed for a 5 cm thick surface layer of the sediment collected over a 50 m river stretch. Before chemical analysis, the samples were mixed; in this way, a so-called average sample was obtained for each river. The measurement technique for HM concentrations in sediments and basic information regarding the quality of the analyses are presented in Table 1 [89].

### 2.3. Preliminary Data Analysis

The preliminary data analysis included the identification of data gaps, concentration values below the detection limit and outlier values. The analysis shows that there were no data gaps in the dataset. The concentrations of Cd, Cu and Pb below the detection limit were replaced with concentrations at half the detection limit for Cd (0.025 mg·kg^−1^), Cu (0.2 mg·kg^−1^) and Pb (0.5 mg·kg^−1^). The Grubbs–Beck test was used to identify outliers. The Shapiro–Wilk test was used to verify whether the HM concentration values have a normal distribution. In addition, descriptive statistics were calculated including minimum, mean, median, maximum and standard deviation values. Violin plots were created to show the concentration variability of individual HMs. Correlation analysis was performed using Spearman’s rank correlation coefficient for initial identification of HM sources. To visualize correlations among HMs, co-occurrence networks were generated by Gephi, WebAtlas (Paris, France) (ver. 0.9.2) [78]. The Grubbs–Beck test and Shapiro–Wilk test and Spearman’s correlation analysis were carried out using STATISTICA 13.1, TIBCO Software Inc. (Palo Alto, CA, USA). All statistical analyses were performed at a significance level of 0.05.

### 2.4. Sediment Contamination and Potential Toxic Effect Assessment

The commonly used enrichment factor (EF) [90], pollution load index (PLI) [91] and metal pollution index (MPI) [92] were used to assess the contamination of river sediments with Cr, Cd, Cu, Zn, Pb and Ni. In order to calculate the EF and PLI index values, the geochemical background values were used, which for Poland are assumed as follows: Cr—5 mg·kg^−1^, Cd—0.5 mg·kg^−1^, Cu—6 mg·kg^−1^, Fe—10,000 mg·kg^−1^, Ni—5 mg·kg^−1^, Pb—10 mg·kg^−1^ and Zn—48 mg·kg^−1^ [93]. Moreover, to calculate the EF values, HMs were normalized concerning Fe concentration in the sediment sample as well as concerning the geochemical background values (GBV). The calculation of EF, PLI and MPI values and classification criteria for sediment contamination are presented in the Appendix C. The results of EF, PLI and MPI are presented against the map of Poland. The consensus-based sediment quality guidelines (SQGs), which included a probable effect concentration (PEC) [94], were used to determine the toxic effects of HMs deposited in sediments on aquatic biota. The PECs are concentrations above which the adverse effects on sediment-dwelling organisms are is expected to occur frequently. The mean PEC quotient (Qm-PEC) was also calculated for each sediment sample. Four ranges of the mean PEC quotient were developed by Long et al. [95] for ranking samples in terms of toxicity incidence. Moreover, the TRI index proposed by Zhang et al. [96] was used to assess the impact of HM concentrations in sediments on aquatic organisms. The threshold effect level (TEC) and the probable effect level (PEC) of HMs determined by MacDonald et al. [94] were used during the TRI calculations.

### 2.5. Spatial Variations of HM Concentrations

The analysis of spatial variation of HMs in river sediments was aimed to divide the sampling points into groups of similar HM concentrations. For this purpose, cluster analysis (CA) was applied. CA analysis was performed using Ward’s method. As a measure of similarity, squared Euclidean distances were used. The Kruskal–Wallis test and Dunn’s test, as a post-hoc procedure, were used to assess differences in HM concentrations between distinguished groups of rivers.

To present the spatial variation of HM concentrations in river sediments across Poland, the global Moran’s *I* and the Getis-Ord $G_{i}^{*}$ statistics were used. The global Moran’s *I* statistic [97] is used to depict the degree of spatial clustering of HM concentrations. The global Moran’s *I* statistic is expressed as [98]:

(1) $$I=\frac{n\sum_{i=1}^{n}\sum_{j=1}^{n}w_{i,j}\left(c_{i}-\bar{c}\right)\left(c_{j}-\bar{c}\right)}{\left(\sum_{i=1}^{n}\sum_{j=1}^{n}w_{i,j}\right)\sum_{i=1}^{n}\left(c_{i}-\bar{c}\right)},$$

where *n* is the number of regions in the study area, *c_i_* is the HM concentration in the *i*th region, *c_j_* is the HM concentration in the *j*th region, $\bar{c}$ is the average concentration of HMs of the studied region, *w_i_*_,*j*_ is the spatial weight matrix, and *i* is not equal to *j*.

Moran’s *I* values fall between −1.0 and +1.0. Values −1.0 and +1.0 indicate strong spatial negative and positive autocorrelation, respectively, while a random distribution exists when the Moran’s *I* value is near to zero. For the global Moran’s *I* analysis, the null hypothesis states that the concentrations of individual HMs are randomly distributed among the study area. To verify the null hypothesis the *Z* statistic was calculated as:

(2) $$Z=\frac{I-E\left(I\right)}{Var{\left(I\right)}^{0.5}},$$

where *E*(*I*) is the expectations of Moran’s *I* and *Var*(*I*) is the variance of Moran’s *I*.

If the *Z* value is in the range of −1.96 to 1.96, we cannot reject the null hypothesis at a 0.05 significance level. Therefore, HM concentrations in river sediments are randomly distributed across Poland. However, when the *Z* value is greater than 1.96, then the *p*-value < 0.05 and we reject the null hypothesis. In this situation, the spatial distribution of high concentration of HMs and/or low concentration is more spatially clustered than would be expected. In other cases where *Z* < 1.96 and *p* < 0.05, we reject the null hypothesis too. In this situation, the spatial distribution of high concentrations and low concentrations of HMs is more spatially dispersed than would be expected.

To identify spatial clusters of high concentrations (hotspots) and low concentrations of HMs (cold spots), the Getis-Ord $G_{i}^{*}$ statistic was used. The local Getis-Ord $G_{i}^{*}$ statistic is given as:

(3) $$G_{i}^{*}=\frac{\sum_{j=1}^{n}w_{i,j}c_{j}-\bar{c_{j}}\sum_{j=1}^{n}w_{i,j}}{{\left(\frac{\sum_{j=1}^{n}{c_{j}}^{2}}{n}-{\bar{c_{j}}}^{2}\right)}^{0.5}{\left(\frac{\left(n\sum_{j=1}^{n}{w_{i,j}}^{2}-{\left(\sum_{j=1}^{n}w_{i,j}\right)}^{2}\right)}{n-1}\right)}^{0.5}},$$

A high *Z*-score and small *p*-value indicate spatial clustering of high individual HM concentrations; conversely, a low negative *Z*-score and a small *p*-value indicate a spatial clustering of low individual HM concentrations, whereas if the *Z*-score is near to 0, this indicates a lack of apparent spatial clustering. The results of the analysis are presented in graphical form with hot and/or cold spots highlighted at 0.01, 0.05 and 0.10 significance levels. The ArcGIS 10.7.1, ESRI (Redlands, CA, USA) software was used to calculate Moran’s *I* and Getis-Ord $G_{i}^{*}$ statistics.

### 2.6. Sediment Contamination and Potential Toxic Effect Assessment

The PCA method was used to identify pollution sources and to link HM content in sediments with catchment land use. The justification for choosing the PCA method for the data is confirmed by the results of detrended correspondence analysis (DCA). The length of the first DCA axis is <3 S.D. (S.D.—standard deviation units), which means that linear ordination methods are preferable [99]. During the PCA analysis, the following land use classes are distinguished: urban fabric (UrF), industrial (In), mine, dump and construction sites (MDC), artificial (Art), arable land (Ara), permanent crops (PrC), pastures (Ps), heterogeneous agricultural areas (HAg), forests (Fo), scrub and/or herbaceous vegetation associations (SaH), inland wetlands (IWe) and inland waters (IWa). Because the HMs had a right-sided (right oblique) distribution, they were transformed using the Box-Cox method. The Box-Cox transformation is used to transform variables in such a way that their distribution, after transformation, has a distribution as close to normal as possible. Finally, the transformed concentration values obtained as a result of the Box-Cox method were scaled to a range from 0 to 1. This procedure was intended to avoid the possibility that the HMs with the highest values would have the greatest influence on the analysis result.

Moreover, to source apportionment of HMs in river sediments, positive matrix factorization (PMF) was used. PMF was developed by Paatero and Tapper [100]. In PMF, the factor analytic model is expressed by the following formula:

(4) X=G·F+E,

The PMF model decomposes the data matrix *X (i* × *j*), which includes *i* observed sampling points and *j* analyzed heavy metals in a pollution source contribution matrix *G* (*i* × *p*), which includes *p* pollution sources, the pollution source profile matrix *F* (*p* × *j*) and the residual matrix *E* (*i* × *j*). The general structure of the PMF model can be expressed as [52]:

(5) $$x_{i,j}=\sum_{p=1}^{n}g_{i,p}f_{p,j}+e_{i,j},$$

where *p* is the number of pollution sources, *g* is the amount of mass contributed by each factor to each individual sample, *f* is the heavy metal profile of each pollution source, and *e* is the residual. Pollution source contributions and pollution source profiles are derived by a model minimizing the objective function *Q*:

(6) $$Q=\sum_{i=1}^{n}\sum_{j=1}^{m}{\left(\frac{x_{i,j}-\sum_{p=1}^{n}g_{i,p}f_{p,j}}{u_{i,j}}\right)}^{2},$$

where *u_i_*_,*j*_ is the uncertainty. Optimum numbers of pollution sources (factors) were found by the value of *Q*, which shows the model fitting capability [101]. The number of significant factors was determined according to the approach presented by Garas et al. [68] and Comero et al. [76].

A global minimum was computed by changing the seed value from 1 to 20 for each model run [102]. In this study, we used PMF model version 5.0 developed by the Environmental Protection Agency (EPA), US [52]. The data input to the PMF model are a matrix of HM concentrations at each sampling point and a matrix of uncertainties associated with the chemical analysis of individual HMs. A detailed method for quantifying the uncertainties is described by Ellison and Williams [103]. To identify the uncertainty σ for individual HMs the following simple methods were used [104,105]:

For concentration data below the detection limit:

(7) $$x_{i,j}=\frac{d_{i,j}}{2},\;\sigma_{i,j}=\frac{5d_{i,j}}{6},$$

For concentration data beyond the detection limit:

(8) $$x_{i,j}=c_{i,j}\;\mathrm{if}\;x_{i,j}\leq 3d_{i,j},\;\sigma_{i,j}=\frac{1}{3}d_{i,j}+0.2\cdot c_{i,j},\;x_{i,j}=c_{i,j}\;\mathrm{if}\;x_{i,j}>3d_{i,j},\;\sigma_{i,j}=\frac{1}{3}d_{i,j}+0.1\cdot c_{i,j},$$

where *x_i_*_,*j*_ is the concentration used in the PMF model, *d_i_*_,*j*_ is the detection limit of each component, *σ_i_*_,*j*_ is the uncertainty of *x_i_*_,*j*_, and *c_i_*_,*j*_ is the measured HM concentration.

## 3. Results

### 3.1. Characteristics of HM Concentrations in River Sediments

Analysis of heavy metal concentrations in river sediments showed that for up to 25 sediment samples Cd concentrations were below the detection limit (DL) (Table 2). Additionally, for Cu and Pb, concentrations in seven samples and one sample were below the DL, respectively. Analysis of the dataset using the Grubbs–Beck test revealed outliers in the dataset. For Cd, Cr and Ni in three samples, the concentrations were at a very high level; based on the Grubbs–Beck test, these values were identified as outliers. Analysis by the Grubbs–Beck test revealed the presence of outliers for Pb and Zn in two sediment samples and Cu in 1 sediment sample. Considering the sampling points, as many as five outliers were identified in the sediments of the Bystrzyca River sediments (No. 4). At this point, concentrations of Cd, Cr, Cu, Ni and Zn were at a very high level. In the sediments of the Parseta River (No. 25), there were three outliers in Cd, Pb and Zn concentrations, respectively. In the sediments of the Rega River (No. 33), Cr and Ni concentrations were considered as outliers. In the sediments of Poprad River (No. 29) Ni concentrations were at a very high level, while Cr concentrations were high in Pasleka River (No. 26), Cd in Bzura River (No. 5) and Pb in Barycz River (No. 1). HM concentrations based on median values can be ordered in the following ascending order Cd < Ni < Cu < Cr < Pb < Zn. This order of HMs relates to that presented within the geochemical background values. The descriptive statistics for the analyzed HMs are shown in Table 2.

**Figure 2:**
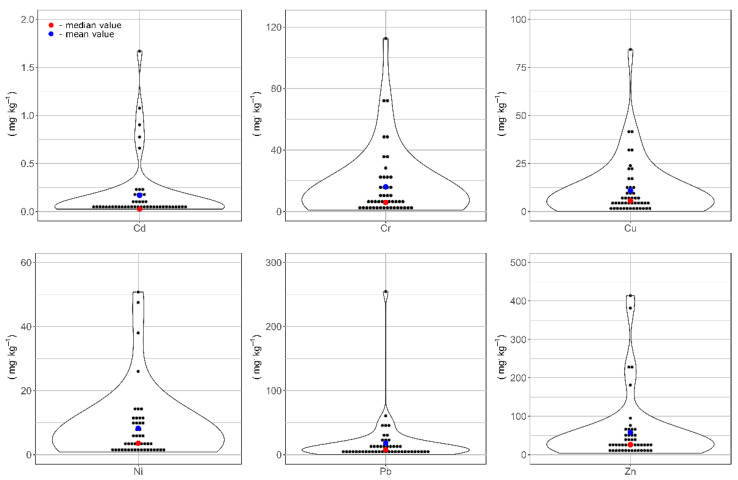
Violin plots showing the concentrations of individual HMs.

Analysis of HM concentrations by the Shapiro–Wilk test showed that their distribution differed from the normal one. In all cases, the concentration distribution was strongly right-skewed. In all cases, the values of the skewness coefficient were higher than 2.5. Moreover, the distribution of analyzed elements is leptokurtic and the values of kurtosis were higher than 3. Right-skewed distribution of HM concentrations is visualized in violin plots (Figure 2). For all HMs, the mean concentration values (blue dots) were higher than the median values (red dots).

**Figure 3:**
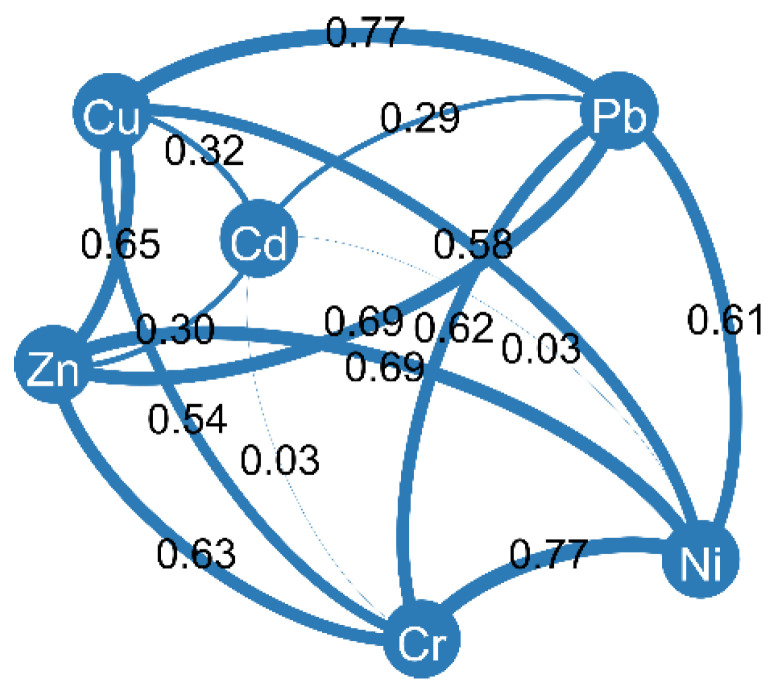
Spearman correlation analysis results.

Correlation analysis performed using Spearman’s test showed a high degree of correlation between the concentrations of Cu, Zn, Pb, Ni and Cr. This may indicate the same type of pollution sources and delivery pathway. Spearman’s correlation coefficient values were generally higher than 0.60 (Figure 3). In contrast, Cd concentrations were not associated with Ni and Cr (correlation coefficient values were 0.03) and relatively weakly correlated with Cu, Pb and Zn (correlation coefficient values ranging from 0.29 to 0.32). This indicates a different pollution source and/or delivery pathway.

### 3.2. Sediment Contamination and Potential Toxic Effects of HMs

**Figure 4:**
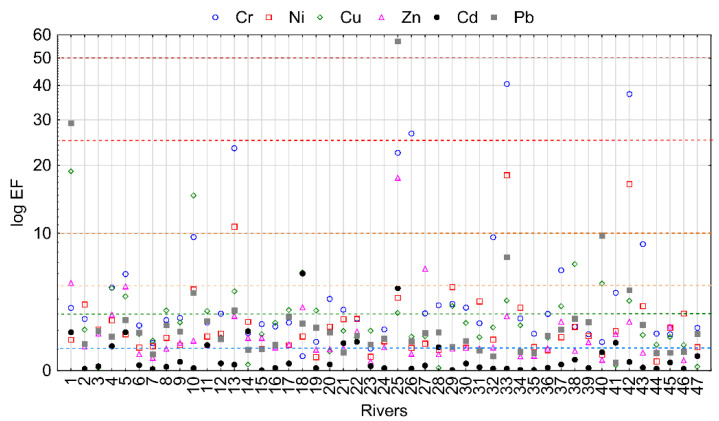
EF value for analyzed HMs. The numbers of sampling points are listed in the Appendix A.

The analysis of river sediments showed their local pollution with HMs. Considering EF values, the weakest pollution occurred for Cd (Figure 4). In 36 rivers, the values indicated no pollution, and in 9 rivers, they indicated minor enrichment. On the other hand, the highest pollution was recorded for Cr. The EF values were higher than 5 in 10 rivers, which indicates more than moderately severe enrichment. For other elements, EF values were higher than 5 in 5, 4, 3 and 3 rivers for Cu, Pb, Zn and Ni, respectively.

**Figure 5:**
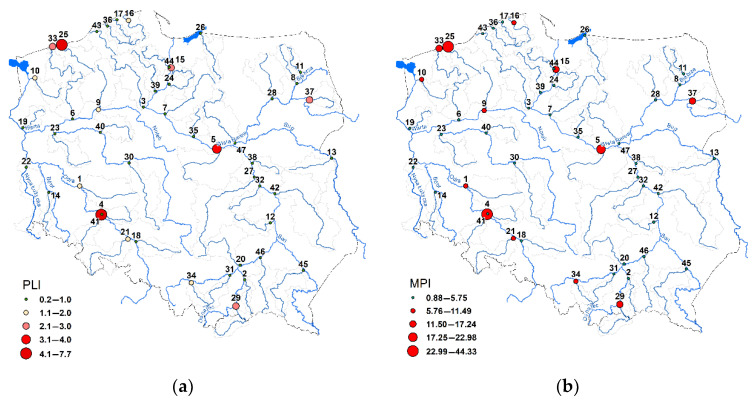
River sediment contamination by HMs based on PLI (**a**) and MPI (**b**) indices. The numbers of sampling points are listed in the Appendix A.

Considering the PLI index, it ranged from 0.15 to 7.71. In 34 rivers, PLI values were lower than 1, which indicates no pollution, while in 13 rivers, sediments were polluted. The highest PLI values were found in sediments of the Bystrzyca (No. 4), Parseta (No. 25) and Bzura (No. 5) rivers, where PLI values were 7.71, 4.71 and 3.05, respectively. PLI values below 0.2 were found in the Kwisa (No. 14), Obra (No. 23), Swider (No. 38), Widawa (No. 41) and Wkra (No. 47) rivers (Figure 5a). The MPI values ranged from 0.88 to 44.22. The MPI results are consistent with the PLI results (Figure 5b). The analogous result of the sediment classification as obtained with the PLI can be obtained based on the MPI with a limit value of 5.75.

**Figure 6:**
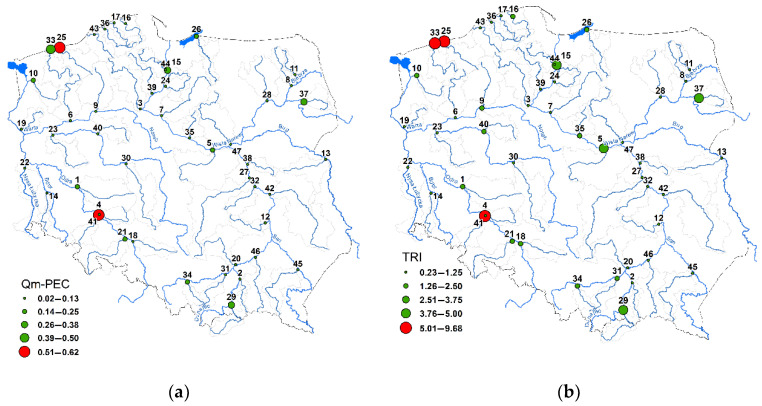
Potential toxic effects of HMs on aquatic organisms based on Qm-PEC (**a**) and TRI (**b**) indices. The numbers of sampling points are listed in the Appendix A.

The analysis of HM concentrations concerning the limit values proposed by MacDonald et al. [94] enables the assessment of their potential toxic effect. It was found that, considering Qm-PEC values, there is a potential toxic effect of HMs on aquatic biota only in the rivers Parseta (No. 25) and Bystrzyca (No. 4) (Figure 6a). The values of Qm-PEC in both cases were 3.7 and 3.6. In 28 rivers the values of Qm-PEC were lower than 0.5. Considering the TRI values, they ranged from 0.23 to 9.68. In three rivers, the TRI values were higher than 5, which indicates the possibility of the potential toxic effect of HMs on aquatic biota. In addition to the previously indicated rivers using the Qm-PEC, it is important to highlight the Rega River (No. 33), in which the TRI value is 5.34 (Figure 6b).

### 3.3. Spatial Variation of HMs Concentrations in River Sediments

**Figure 7:**
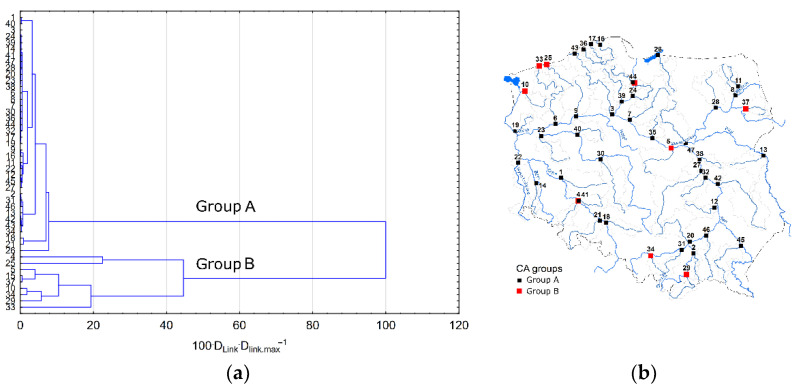
Results of cluster analysis (**a**) and their spatial projection against Poland (**b**). The numbers of sampling points are listed in the Appendix A.

The analysis of HMs’ spatial variation was started by dividing the rivers into groups using the CA methods. Thus, two groups, A and B, were distinguished (Figure 7). Group A included 38 rivers and group B included 9 rivers (Figure 7a). The comparison of HM concentrations in distinguished groups using the Mann–Whitney test showed that Cr, Cu, Ni, Pb and Zn concentrations in group B are higher than those in group A. The differences are significant at the 0.05 level. Regarding Cd, there are no significant differences between the distinguished groups. Spatial projection of CA results indicates the dispersion of rivers in group B across the whole territory of Poland (Figure 7b).

On the other hand, analysis of HM concentrations using Moran’s *I* statistic showed that there is no clustering tendency by individual HMs in the analysis area. This indicates that there is no regional similarity of HM concentrations in the river sediments. For Cd, Cr and Ni, a random distribution is observed, while for Cu, Pb and Zn, a dispersed distribution is observed (Table 3). The results show that there are no clear regional sources of pollution, while the dispersed pattern indicates the presence of local/point sources of pollution.

**Figure 8:**
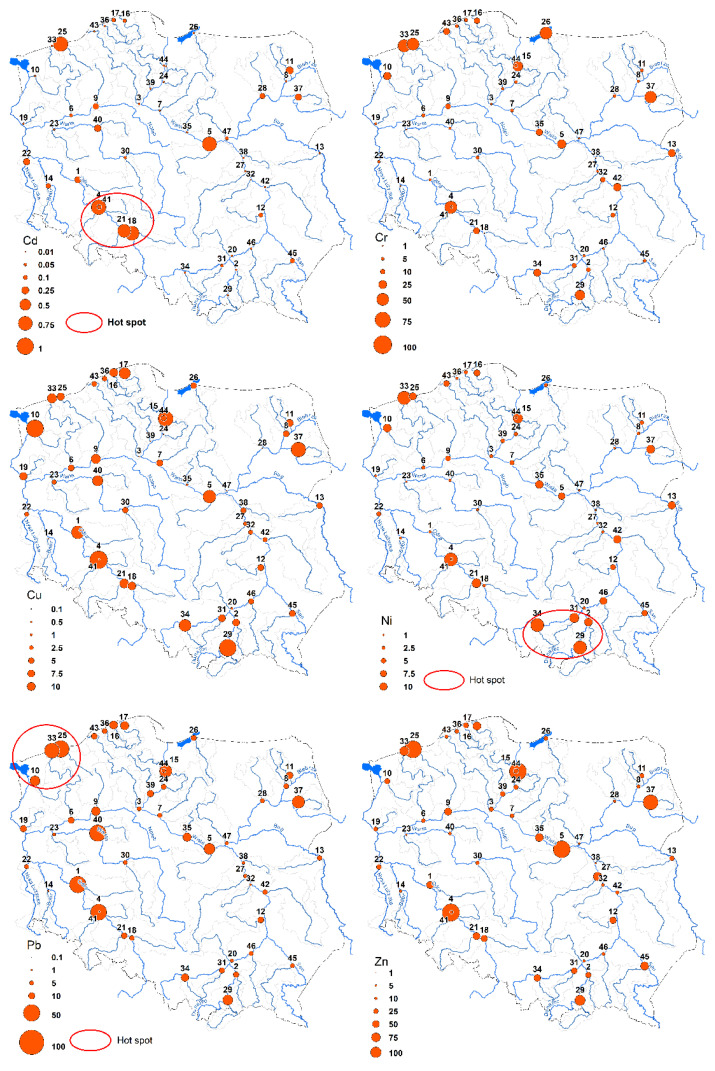
Hotspot locations of Cd, Ni and Pb concentrations based on the Getis-Ord $G_{i}^{*}$ statistic. The numbers of sampling points are listed in the Appendix A.

The analysis using the Getis-Ord $G_{i}^{*}$ statistic shows the hotspots for Cd, Ni and Pb (Figure 8). In the case of Cd and Ni, hotspots appeared, respectively, in the southwestern and southern parts of Poland and Pb in northwestern Poland. These results indicate that there is a great diversity of pollution sources and pathways of HM delivery to surface waters.

### 3.4. Pollution Source Identification

**Figure 9:**
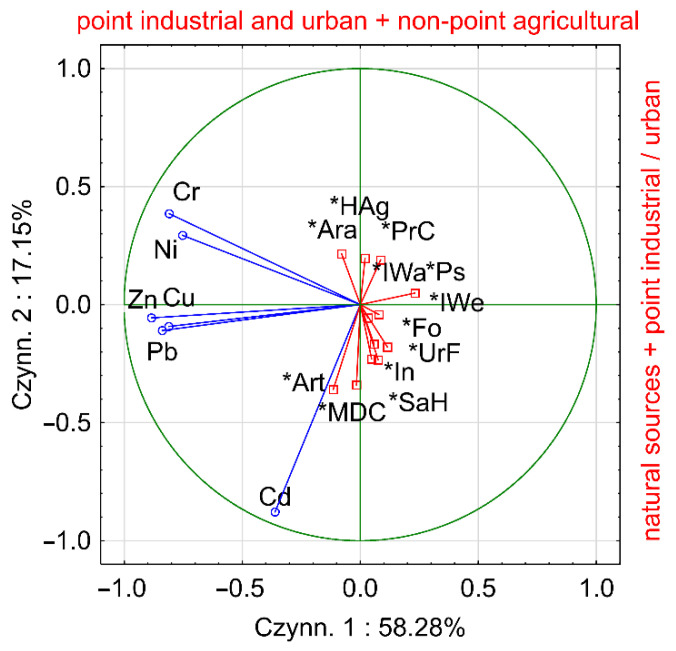
PCA results.

PCA analysis distinguished two significant factors, PC1 and PC2, for which the eigenvalues were higher than 1. The distinguished factors explain 58.3% and 17.2% of the original data structure, respectively. The value of KMO analysis confirmed the sampling dataset was adequate. The total contribution of the distinguished factors is 75.5% Cr, Cu, Pb, Ni and Zn were strongly negatively correlated with PC1, while Cd concentrations were negatively correlated with PC2. On the other hand, no correlation was observed between the distinguished factors PC1 and PC2 and the indices characterizing the land use pattern within the catchments (Figure 9). While trying to attribute the distinguished factors PC1 and PC2 to the physical source of supply, the concentrations of HMs should be analyzed against the geochemical background values. For Cd concentrations, it was noted that only in five cases were they higher than the geochemical background values. Thus, the PC2 component can be generally considered as a natural source of this element in river sediments, disturbed by point sources of pollution. On the other hand, Cr, Cu, Pb, Ni and Zn concentrations exceeded geochemical background values more frequently, including 14 times for Zn and 22 times for Cr. In addition, considering the lack of correlation between PC1 and land use structure, it can be assumed that the transport of these elements is related to point sources (industrial and urban pollution) and possibly to non-point pollution sources (fertilizer use in agriculture). It should be noted that the Cr and Ni sources are slightly different than those of Cu, Pb and Zn.

**Figure 10:**
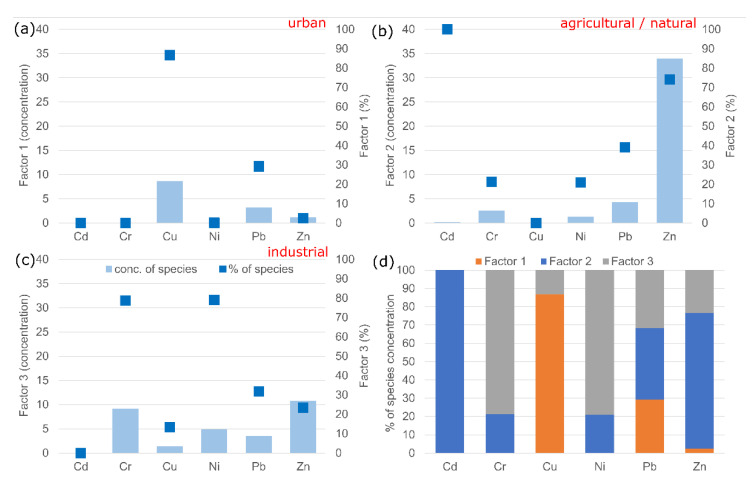
Source apportionment (**a**–**c**) and source contribution (**d**) to HM supply obtained by the PMF method.

Slightly different results were obtained using the PMF method. Three important factors associated with HMs delivery to sediments were identified. Overall, Factor 1 is responsible for 15.2% of HMs concentrations in sediments. Factors 2 and 3 contribute to the remaining pool of HMs, 49.8% and 34.9%, respectively. On the other hand, the analysis of the contribution of factors to the delivery of individual HMs showed that factor 1 is mainly associated with Cu delivery (86.7%), lower Pb delivery (29.2%), as well as minimal Zn delivery (2.5%) (Figure 10a). Factor 2 is mainly responsible for the delivery of Cd (100%) and Zn (74.1%) and to a lower extent Pb (39.1%), Cr (21.3%) and Ni (21.0%) (Figure 10b). In contrast, Factor 3 is mainly responsible for Ni (79.0%) and Cr (78.7%) in river sediments and to a lower extent Pb (31.7%), Zn (23.4%) and Cu (13.3%) (Figure 10c). In order to attribute a specific pollution source to each factor, the pollution profiles of urban, industry and agriculture were analyzed. Analysis of individual pollution profiles showed very high inter- and intra-group variability (Figure 10d). Taking into account the pollution profiles of individual sources, catchment land cover structure and land cover structure above the sampling points, the most likely sources of HMs delivery to sediments were identified. Considering the contribution of the distinguished factors to the delivery of individual HMs, it should be pointed out that Factor 1 can be associated with urban pollution. Factor 2 suggested the combined impact of agriculture and natural sources. The values most strongly associated with Factor 2, Cd and Zn, were higher than the geochemical background values in only 5 and 14 cases, respectively. On the other hand, Factor 3 can be associated with industrial pollution. In general, Factor 3 is responsible for the delivery of mainly Cr and Ni, whose concentrations exceeded the geochemical background values in 22 and 21 cases, respectively.

## 4. Discussion

In this study, HM concentrations and ecological risk were evaluated for 47 rivers located in Poland. Most previous studies were mainly related to the concentrations of rivers, lakes and reservoirs analyzed at the local and regional scale [15,18,19,20,22,24,31,32,33,34,35]. There is a lack of systematic study in the literature considering the analysis of HM concentrations for rivers located in nearly the whole area of the country. Analysis of a bigger group of rivers can help to understand the relationship between HM concentration and localization. In this study, Getis-Ord $G_{i}^{*}$ statistical analysis demonstrated that in the case of Cd and Ni, hotspots appeared respectively in the southwestern and southern parts of Poland and Pb in northwestern Poland. Finding group sources of contamination is very important to find new methods in water environment protection at a regional scale.

Anthropogenic and natural pollution are two main sources of pollution in a water environment. Most previous studies suggest that HM contamination is mainly connected with anthropogenic sources [55,106,107]. Cheng et al. [106] reported that Pb contamination was mainly from natural background and anthropogenic sources, with contribution rates of 45% and 55%, respectively. Results obtained in this study confirm it; according to the PMF method, 29.2% of Pb pollution is connected with urban sources and 39.1% with agricultural and natural sources. Setia et al. [107] highlighted that non-point sources, such as agriculture surface runoff from urban areas and soil erosion, have an impact on HM contamination and should not be ignored due to their complexity and difficult analysis. The PMF analysis in this study shows that agriculture and natural sources are mainly responsible for the supply of most HMs (Cd 100%, Zn 74.1%, Pb 39.1%, Cr 21.3% and Ni 21.0%). In turn, the PCA analysis in this study identified that Cr, Cu, Ni, Pb and Zn reach waters and sediments from point and non-point sources, while Cd primarily originates from natural and point sources.

The present results confirm the results from previous studies, showing that HM concentrations are mainly connected with point sources of pollution [6,23,41]. An analysis of 47 rivers in Poland shows that there are two main sources of point pollution, industrial and urban. Setia et al. [107] suggested that the discharge of chemical waste from industrial areas is one of the major sources of contamination in the aquatic environment. PMF analysis in this study shows that Factor 3 associated with industrial pollution is responsible for the delivery of mainly Cr and Ni. Additionally, HM contamination is mainly connected with urban and industrial discharges including effluents from chemicals, pharmaceuticals, electroplating, steel and textile manufacturing units as major sources among other industries. The PMF method in this study shows that Factor 1, connected with urban sources of pollution, is mainly associated with Cu (86.7%), Pb (29.2%) and Zn supply (2.5%). Additionally, according to Haghnazar et al. [108], river morphology and hydraulic parameters regulate sediment transport and other processes which have an impact on the accumulation of HMs in sediments.

Depending on the pollution source, the HM contamination in water and sediments varies in space and time [109]. Moreover, the pattern of HM concentrations in sediments is an individual feature of the basin. In natural basins, the contribution of particular HMs is influenced by natural processes related to weathering of bedrock, but in most basins, the main factor influencing HM concentrations is the character of point pollution sources. HM concentrations based on median values can be ordered in the following ascending order: Zn > Pb > Cr > Ni > Cd. These results confirm previous research for the Oder, Vistula and Warta rivers, where Zn was characterized by the highest concentration and Cd the lowest [22,45].

Multivariate statistics applied in this study allow us to define the main pollution sources, but it is also important to use a tool that allows one to assess the spatial distribution of pollutants. HM concentrations can be better visualized with a geographic information system (GIS) [22,45]. The use of Moran *I* and Getis-Ord $G_{i}^{*}$ methods allows us to determine the spatial pattern of HM contents in river sediments. The results thus obtained allow identification of potential points as well as area (regional) sources of pollution. Similar results were obtained using the CA method in combination with spatial presentation of clustering results in a GIS environment. The methodology applied in this study can also be applicable in other climatic zones and types of waters. The main limitation of the analysis may be the interpretation of the PCA and PMF results. In particular, the assignment of specific sources of pollution to the distinguished factors requires more extensive expert knowledge, considering the main pollution sources. Moreover, considering that nowadays in each catchment there is generally an interaction of many different pollution sources, which is modified by the fluvial processes, for certain rivers (more polluted), a detailed analysis is necessary as regards the location of point sources of pollution, specificity of pollution discharged to waters, fertilizer consumption and other factors. Moreover, as shown in this study, there is no clear relationship between the main land-use types and the HM concentrations. Future studies should address more detailed land-use types and analyze their pattern within the catchment, river floodplain valley or even on the river shoreline.

The results obtained in this study confirm previous studies that HMs may pose ecological and human health risk [110,111,112]. The main exposure pathways from surface water to humans are water ingestion, water used to agricultural irrigation and direct contact with water during recreation activities [113]. According to Hoang et al. [113], HMs adverse effects on human health include cancer, anemia and diabetes. It is important to use novel techniques to analyze changes in HMs pollution, sources and spatial distribution to protect the environment and human health.

## 5. Conclusions

The findings obtained from the analyses made it possible to formulate the following specific conclusions:

- The pattern of HM concentrations in the sediments of 2/3rd of the rivers refers to the concentration pattern resulting from the geochemical background. The contamination of sediments of 1/3rd of the rivers shows a change in the natural pattern of HM concentrations and their higher values above the geochemical background values.
- The values of geochemical indices EF, PLI and MPI indicate sediment pollution in 1/3rd of the analyzed rivers. The identified points with higher HM concentrations were dispersed over the whole area of Poland.
- Only single rivers in Poland were detected where HMs may have toxic effects on aquatic biota.
- Visualization of cluster analysis results on the background of Poland indicates a lack of non-point sources of pollution resulting, e.g., from areas of very intensive agriculture and industrial activity and low development of water and sewage systems.
- Spatial autocorrelation analysis of HM concentrations in river sediments using the Moran *I* method indicates a random and dispersed pattern. This indicates the presence of local point sources of pollution that overlap with the delivery of HMs from natural sources.
- Analysis using the Getis-Ord $G_{i}^{*}$ test indicated the presence of hotspots of Cd, Ni and Pb. These locations require a detailed source analysis first.
- The PCA analysis identified two sources of HM delivery to the aquatic environment. The main pool of Cr, Cu, Ni, Pb and Zn reaches waters from point and surface sources, while Cd concentrations have dominant origins from natural and point sources.
- The PMF analysis has quantitatively identified three sources of pollution. Among them, urban pollution is mainly responsible for Cu delivery, agricultural pollution for Zn delivery and industrial pollution for Ni and Cr delivery.
- The analysis showed no relationship between catchment land-use patterns and HM contents in river sediments.

## Figures and Tables

**Table 1:** Chemical analysis technique and qualitative results [89].

Element	Analysis Technique	Detection Limit (mg·kg^−1^)	Uncertainty of Analysis (%)
Cd	ICP-OES	0.05	15
Cr	ICP-OES	0.30	20
Cu	ICP-OES	0.40	20
Ni	ICP-OES	0.40	15
Pb	ICP-OES	1.00	15
Zn	ICP-OES	0.50	20

ICP-OES—inductively coupled plasma—optical emission spectrometry.

**Table 2:** Summary statistics of heavy metal concentrations (mg·kg^−1^) in river sediments.

Statistics	Cr	Ni	Cu	Zn	Cd	Pb
Minimum	0.854	0.791	0.200	4.41	0.025	0.50
Mean	16.1	8.21	10.92	57.9	0.168	18.0
Median	5.88	3.58	5.56	26.0	0.025	7.59
Maximum	113	50.8	84.30	414	1.670	255
Standard deviation	22.1	11.0	14.96	86.9	0.321	37.4
Coefficient of variation	1.38	1.34	1.37	1.50	1.90	2.07
Skewness	2.61	2.69	3.03	2.97	3.19	5.62
Kurtosis	7.68	7.34	11.6	8.8	10.8	35.0
Number of values below DL	0	0	7	0	25	1
Number of outlier values	3	3	1	2	3	2
GBV	5.0	5.0	6.0	48.0	0.50	10.0

**Table 3:** Results of autocorrelation analysis based on Moran’s *I* statistic.

Parameters	Moran *I*		
	*p*-Value	Z-Score	Pattern
Cd	0.741	0.33	random
Cr	0.147	−1.45	random
Cu	0.007	−2.70	dispersed
Ni	0.045	−2.00	dispersed
Pb	0.073	−1.79	random
Zn	0.020	−2.32	dispersed

## Data Availability

Data sharing is not applicable to this article.

## Appendix A. Summary of the Sampling Points and River Names

**Sampling Point**	**River Name**	**Sampling Point**	**River Name**
1	Barycz	25	Parsęta
2	Biała	26	Pasłęka
3	Brda	27	Pilica
4	Bystrzyca	28	Pisa
5	Bzura	29	Poprad
6	Drawa	30	Prosna
7	Drwęca	31	Raba
8	Ełk	32	Radomka
9	Gwda	33	Rega
10	Ina	34	Skawa
11	Jegrznia	35	Skrwa
12	Kamienna	36	Słupia
13	Krzna	37	Supraśl
14	Kwisa	38	Świder
15	Liwa	39	Wda
16	Łeba	40	Wełna
17	Łupawa	41	Widawa
18	Mała Panew	42	Wieprz
19	Myśla	43	Wieprza
20	Nida	44	Wierzyca
21	Nysa Kłodzka	45	Wisłok
22	Nysa Łużycka	46	Wisłoka
23	Obra	47	Wkra
24	Osa		

## Appendix B. Catchment Characteristics

**Sampling Point**	**Area [km^2^]**	**Perimeter [km]**	**Mean Elevation** **[m a.s.l]**	**Mean Slope [%]**	**Drainage Density [km/km^2^]**	**Artificial Surfaces (%)**	**Agricultural Areas (%)**	**Forest and Seminatural Areas (%)**	**Wetlands (%)**	**Water Bodies (%)**
1	5543.21	465.03	122.04	0.72	0.64	6.63	52.92	38.36	0.10	1.99
2	976.06	255.52	373.17	7.41	0.72	4.90	64.80	30.24	0.00	0.06
3	4656.88	661.32	130.67	1.43	0.35	4.06	43.31	49.56	0.17	2.89
4	1778.79	303.92	289.58	3.76	0.71	6.22	79.03	14.39	0.00	0.36
5	7718.63	593.22	126.12	0.73	0.49	9.06	71.27	19.18	0.11	0.37
6	3287.91	478.59	101.15	1.74	0.31	1.40	32.32	63.62	0.08	2.59
7	5694.52	712.11	119.85	1.70	0.39	6.29	55.43	32.15	0.72	5.40
8	1555.78	376.03	142.25	1.54	0.42	1.97	70.95	23.70	0.48	2.90
9	4958.89	579.38	133.55	1.22	0.36	3.31	44.58	49.45	0.18	2.49
10	2141.55	412.66	64.18	1.33	0.44	4.55	64.13	29.41	0.16	1.75
11	1061.66	293.02	141.45	1.04	0.48	4.62	31.62	45.79	8.98	8.99
12	2021.17	329.35	247.69	2.37	0.42	11.00	54.65	33.94	0.00	0.40
13	3281.87	630.77	153.24	0.40	0.55	3.82	71.64	24.04	0.12	0.39
14	1022.75	273.75	302.12	3.21	0.66	10.06	44.30	45.24	0.06	0.34
15	969.03	248.36	71.16	1.54	0.35	1.92	79.38	17.48	0.33	0.90
16	1089.00	355.46	72.88	2.17	0.41	2.62	48.69	43.72	0.84	4.12
17	809.20	343.51	102.80	1.77	0.40	2.92	60.02	32.63	0.25	4.17
18	2113.23	298.41	234.22	0.76	0.53	6.77	38.75	53.29	0.05	1.14
19	1301.15	291.29	64.85	1.06	0.36	3.09	54.81	39.31	0.28	2.50
20	3844.27	434.80	261.00	1.95	0.41	11.02	57.21	31.11	0.11	0.55
21	4485.86	539.00	364.27	4.64	0.67	9.78	40.92	47.13	0.14	2.03
22	4082.06	582.50	222.56	4.03	0.38	6.84	40.93	51.49	0.11	0.62
23	2759.35	481.89	75.51	0.95	0.37	3.78	50.83	43.57	0.19	1.63
24	1601.68	333.41	94.31	1.72	0.37	3.81	65.53	26.63	0.88	3.15
25	3068.68	460.47	86.54	1.77	0.47	3.01	52.41	44.13	0.06	0.40
26	2316.67	526.86	100.36	1.94	0.59	2.88	55.95	39.07	0.29	1.81
27	9252.48	975.52	214.48	1.13	0.40	4.77	59.22	35.25	0.13	0.63
28	4513.67	565.83	133.01	1.23	0.37	2.34	44.31	43.70	1.14	8.51
29	2082.48	298.69	648.84	13.86	0.23	5.23	38.33	56.23	0.03	0.19
30	4914.76	622.88	152.21	0.80	0.42	5.23	76.33	18.21	0.07	0.15
31	1530.22	281.77	439.38	8.58	0.83	6.91	55.98	36.37	0.00	0.74
32	2107.93	319.40	178.50	0.95	0.48	12.46	58.14	28.70	0.06	0.64
33	2738.58	486.52	73.74	1.48	0.45	4.69	59.43	34.03	0.17	1.68
34	1175.67	219.93	506.47	9.37	0.84	6.95	42.53	48.81	0.00	1.72
35	1665.54	316.38	120.19	0.61	0.53	1.01	86.18	12.57	0.12	0.13
36	1597.50	404.38	114.26	2.32	0.42	4.79	45.43	48.30	0.02	1.46
37	1843.73	290.00	155.78	1.58	0.35	6.57	39.36	53.55	0.31	0.20
38	1157.86	248.07	151.48	0.80	0.53	8.25	64.41	27.08	0.05	0.21
39	2325.60	533.48	121.63	1.41	0.34	3.57	54.94	37.74	0.18	3.57
40	2620.52	439.74	97.65	0.78	0.45	3.56	72.06	22.49	0.12	1.77
41	1741.38	248.20	160.57	0.74	0.64	7.83	67.12	24.82	0.00	0.23
42	10,453.85	1030.29	199.31	1.52	0.39	6.74	68.95	23.12	0.21	0.98
43	1534.03	360.46	88.66	1.96	0.46	2.79	47.54	48.36	0.13	1.18
44	1606.87	347.06	125.91	1.94	0.41	5.09	66.77	25.74	0.15	2.25
45	3529.28	478.09	297.19	4.73	0.66	12.06	58.02	29.76	0.00	0.16
46	4099.78	572.82	334.06	5.29	0.65	6.19	54.93	38.54	0.01	0.33
47	5341.13	510.27	130.32	0.80	0.40	4.40	73.03	21.93	0.47	0.17

## Appendix C. List of Geochemical Indices Used in This Study

**Index**		**Formula**	**Classification**	**Source**
Enrichment Factor	EF	EF*_i_* = (C_i_/C_Fe_)/(B_i_/B_Fe_) where: *C*_i_—the concentration of HM in the sediments (mg·kg^−1^) C_Fe_—the concentration of iron (Fe) B_i_—the reference geochemical background value of each HM B_Fe_—the reference geochemical background value of iron (Fe)	EF ≤ 1 no enrichment 1 < EF ≤ 3 minor enrichment 3 < EF ≤ 5 moderate enrichment 5 < EF ≤ 10 moderately severe enrichment 10 < EF ≤ 25 severe enrichment 25 < EF ≤ 50 very severe enrichment EF > 50 extremely severe enrichment	Ergin et al. (1991) [90]
Pollution Load Index	PLI	PLI = (CF_i__1_ × CF_i__2_ × … × CF_in_)^1^^/n^ where: n—the number of HMs CF—contamination factor defined for each studied HM	PLI < 1 no pollution PLI ≥ 1 pollution	Tommilson et al. (1980) [91]
Metal Pollution Index	MPI	MPI = (C_i__1_× C_i__2_ × … × C_in_)^1^^/n^ where: C_i_—the concentration of HM in the sediments (mg·kg^−1^) n—the number of considered HMs	MPI < 1 no pollution MPI ≥ 1 pollution	Usero et al. (1997) [92]
Mean PEC quotient	Q_m-PEC_	Q_m-PEC_ = ∑*_ni_*_=__1_ (C_i/_PEC_i_) where: C_i_—the concentration of HM in the sediments (mg·kg^−1^) PEC_i_—the probable effect concentration of each HM	Q_m-PEC_ < 0.5 not toxic Q_m-PEC_ ≥ 0.5 toxic	MacDonald et al. (2000) [94]
Toxic Risk Index	TRI	TRI = ∑*_ni_*_=__1_TRI_i_ = {[(C_i_/TEC_i_)^2^ + (C_i_/PEC_i_)^2^]/2}^1^^/2^ where: n—the number of HMs C_i_—the concentration of HM in the sediments (mg·kg^−1^) TEC_i_—the threshold effect concentration of each HM PEC_i_—the probable effect concentration of each HM TRI_i_—the toxic risk index of each HM	TRI ≤ 5 no toxic risk 5 < TRI ≤ 10 low 10 < TRI ≤ 15 moderate 15 < TRI ≤ 20 considerable 20 < TRI very high	Zhang et al. (2016) [96]
